# *EFG1*, Everyone’s Favorite Gene in *Candida albicans*: A Comprehensive Literature Review

**DOI:** 10.3389/fcimb.2022.855229

**Published:** 2022-03-22

**Authors:** Virginia E. Glazier

**Affiliations:** Department of Biology, Niagara University, Niagara Falls, NY, United States

**Keywords:** EFG1, *Candida albicans*, transcription factor regulatory network, biofilm, virulence, transcription factor, fungal pathogen

## Abstract

*Candida* sp. are among the most common fungal commensals found in the human microbiome. Although *Candida* can be found residing harmlessly on the surface of the skin and mucosal membranes, these opportunistic fungi have the potential to cause superficial skin, nail, and mucus membrane infections as well as life threatening systemic infections. Severity of infection is dependent on both fungal and host factors including the immune status of the host. Virulence factors associated with *Candida* sp. pathogenicity include adhesin proteins, degradative enzymes, phenotypic switching, and morphogenesis. A central transcriptional regulator of morphogenesis, the transcription factor Efg1 was first characterized in *Candida albicans* in 1997. Since then, *EFG1* has been referenced in the *Candida* literature over three thousand times, with the number of citations growing daily. Arguably one of the most well studied genes in *Candida albicans, EFG1* has been referenced in nearly all contexts of *Candida* biology from the development of novel therapeutics to white opaque switching, hyphae morphology to immunology. In the review that follows we will synthesize the research that has been performed on this extensively studied transcription factor and highlight several important unanswered questions.

## Introduction

As an obligate human commensal, *Candida albicans* must be able to readily adapt to the niches present in the human host, as well as transition to a pathogenic state under a specific set of environmental cues. Transcriptional programs coordinate the morphology, nutrient absorption, cell wall components, growth/reproduction, and metabolic patterns (among many other traits) employed in response to the combination of environmental cues in a given niche (Rodriguez et al., 2020). These transcriptional patterns are regulated by networks of transcription factors, with a given transcription factor often having both overlapping and distinct functions from the other transcription factors within the network. Efg1 is a transcription factor that has been implicated in several different transcription factor regulatory networks including white/opaque switching, cell morphology, and biofilm formation, all integral to the ability of *C. albicans* to exist as either a commensal or transition to a pathogenic state (Lassak et al., 2011; Nobile et al., 2012; Hernday et al., 2013; Rodriguez et al., 2020; Witchley et al., 2021). As such, understanding the function of *EFG1* is vital to understanding the transcriptional programs that govern virulence in *C. albicans*. This review begins with the structure, binding, and activation of Efg1, then discusses the role of *EFG1* in filamentation, biofilm formation, white/gray/opaque switching, gut colonization, and virulence. For each of these components there is an emphasis on how *EFG1* expression and Efg1 activity are regulated, the transcripts that Efg1 binds, and the interacting protein partners that make up the complex transcription factor regulatory networks that contribute to the success of *C. albicans* as both a commensal and a pathogen.

## Efg1 Structure and DNA Binding Activity

Efg1 is a member of the basic helix-loop-helix (bHLH) transcription factor family. bHLH transcription factors are found throughout the eukaryotic kingdom and are characterized by the presence of a DNA-binding basic region and a helix-loop-helix motif (Jones, 2004). Efg1 belongs to a subcategory of bHLH transcription factors in fungi called APSES (Asm1, Phd1, Sok2, Efg1 and StuA) family transcriptional regulators. APSES proteins can act as either transcriptional activators or repressors and have been shown to regulate several fungal cellular processes including sporulation, mating, aspects of metabolism, and morphology. The ASPES region of the protein contains a highly conserved region of approximately 100 amino acids, the central domain of which forms the bHLH structure (Zhao et al., 2015). Based on targeted deletion analysis in *C. albicans*, this ASPES structure is required for normal yeast morphology, hyphal induction, opaque to white switching, chlamydospore formation, binding to MCB motifs, repressor activity, and for maintaining Efg1 protein levels in the cell (Noffz et al., 2008). Outside of the ASPES region the N- and C- terminal regions also have specific roles in contributing to chlamydospore formation, opaque to white switching, hyphae formation in Lee’s medium, repressor activity and Czf1 binding (Noffz et al., 2008). The effects observed in mutations to regions outside of the DNA binding region may be due to changes in either protein interaction regions and/or phosphorylation sites. Yeast two-hybrid studies have shown that Efg1 is capable of interacting with several proteins including Czf1, Flo8, and members of the NuA4 histone acetyltransferase complex (Giusani et al., 2002; Cao et al., 2006; Lu et al., 2008; Noffz et al., 2008). These same studies demonstrated that the typical protein-protein interaction site, the APSES structure, is not required for Efg1 protein-protein interactions with Czf1 or Flo8 (Cao et al., 2006; Noffz et al., 2008). Instead Efg1 protein-protein interactions are likely mediated by prion-like domains (PrLDs) found in the N- and C- terminal regions of Efg1 (Frazer et al., 2020). PrLDs have been identified in several transcription factors known to form a transcriptional regulatory network with Efg1, this list includes Czf1 which has been demonstrated to physically interact with Efg1 (Giusani et al., 2002; Frazer et al., 2020). These PrLDs allow for Efg1 and other transcription factors containing PrLDs to undergo liquid-liquid phase separation into larger transcription factor regulatory complexes similar to the super-enhancers found in mammalian systems (Frazer et al., 2020). The ASPES region is also typically associated with homodimer formation in other ASPES transcription factors. Yeast-two hybrid assays of Efg1 suggested Efg1 does not form a homodimer, nor does Efg1 form a heterodimer with the closely related Efh1 transcription factor (Doedt et al., 2004). However phase separation experiments demonstrated that purified Efg1 protein is able to form droplets in a manner dependent on both the N- and C- terminal PrLDs, suggesting that Efg1 is able to undergo liquid-liquid phase separation to form larger condensates of Efg1 complexes with itself (Frazer et al., 2020). It is possible that attachment of LexA to Efg1 in previous yeast-two hybrid experiments may have disrupted the ability of the Efg1 PrLD regions to form multivalent functional assemblies of Efg1.

Several studies have attempted to identify the DNA target sequence of Efg1. Initial studies characterized Efg1 as capable of binding the E Box motif (CANNTG) using an *E. coli* expression system (Leng et al., 2001). Efg1 has also been shown to bind MluI (MCB) in a yeast one-hybrid assay (Noffz et al., 2008). Chromatin immunoprecipitation microarray (ChIP-chip) binding experiments at 30°C in YPD yielded a consensus element of (TATGCATA) with an observed transformation in the binding sequence following hyphae induction in serum at 37°C (Lassak et al., 2011). Subsequent ChIP-chip experiments of Efg1 under biofilm formation conditions and during standard mid-log phase growth generated *cis-*regulatory sequence motifs of (RTGCATRW) and (TGCAT) respectively (Nobile et al., 2012; Hernday et al., 2013). Recruitment of Efg1 to alternative DNA binding sites under different conditions is likely the result of changes in Efg1 phosphorylation status and/or interactions with other transcription factors (Lassak et al., 2011). *In vitro* studies using recombinant Efg1 eliminate the potential for interactions with other DNA binding proteins to modulate the consensus element of Efg1. Use of recombinant Efg1 and the MITOMI 2.0 technique which uses an 8-mer DNA sequence library to screen for the binding affinity identified an Efg1 DNA binding motif of CATGCGY (Hernday et al., 2013).

Efg1 has been observed to be heavily phosphorylated under yeast growth conditions, with the phosphorylation status of Efg1 changing under both hyphal induction and hypoxic conditions (Lassak et al., 2011; Saputo et al., 2014; Desai et al., 2015). Studies using epistasis modeling and phosphomimetic mutations point to residues T208 and T181 in Efg1 as predicted phosphorylation sites for PKA and Cdc28-Hgc1, respectively (note that residues T208 and T181 in assembly 22 SC5314 were previously reported as T206 and T179) (Bockmühl and Ernst, 2001; Bockmühl et al., 2001; Wang et al., 2009). However, changes in the phosphorylation status of Efg1 in a PKA deletion mutant were not detected by western blot of Efg1 under hyphal induction conditions with 10% serum (Lassak et al., 2011). Additionally, phosphoproteomic studies have failed to identify these phosphorylation sites when filamentation was induced with Lee’s medium or GlcNac. Instead, phosphoproteomic studies identified S355, S357, T512, and S518 as Efg1 phosphorylation sites (Willger et al., 2015; Cao et al., 2017; Min et al., 2021). Further investigation into the differences in phosphorylation patterns under different conditions and the potential inconsistency between studies using epistasis and phosphomimetic mutations versus phosphoproteomic techniques are necessary.

## Role of Efg1 in Filamentation and Morphogenesis

As part of the ASPES family of transcription factors, Efg1 is highly homologous to both Sok2 and Phd1, transcription factors which have roles in pseudohyphal growth in *Saccharomyces cerevisiae* (Stoldt et al., 1997). Phd1 is a positive regulator of pseudohyphae formation, binding to the promoter of *FLO11* which is required for the cell-to cell-adhesion that facilitates invasive pseudohyphal growth (Pan and Heitman, 2000; Cullen and Sprague, 2012). Sok2 acts as a negative regulator of pseudohyphae formation repressing expression of both *PHD1* and *FLO11* (Pan and Heitman, 2000; Cullen and Sprague, 2012; Mayhew and Mitra, 2014). Both Sok2 and Phd1 function downstream of the cAMP/PKA pathway in *Saccharomyces cerevisiae* (Cullen and Sprague, 2012; Raithatha et al., 2012). *EFG1* was initially characterized by Joachim Ernst’s group in 1997 through a *Saccharomyces cerevisiae* screen for *C. albicans* genes that promoted pseudohyphal morphogenesis (Stoldt et al., 1997). In this study high levels of Efg1 in *S. cerevisiae* produced robust pseudohyphae, while overexpression of *EFG1* in *C. albicans* caused increased pseudohyphal growth. Conversely, repression of *EFG1* in *C. albicans* produced elongated cells resembling the opaque cell type. As such, these experiments were the first to link *EFG1* to not only cell morphology but also white/opaque switching (Stoldt et al., 1997).

**Figure 1:**
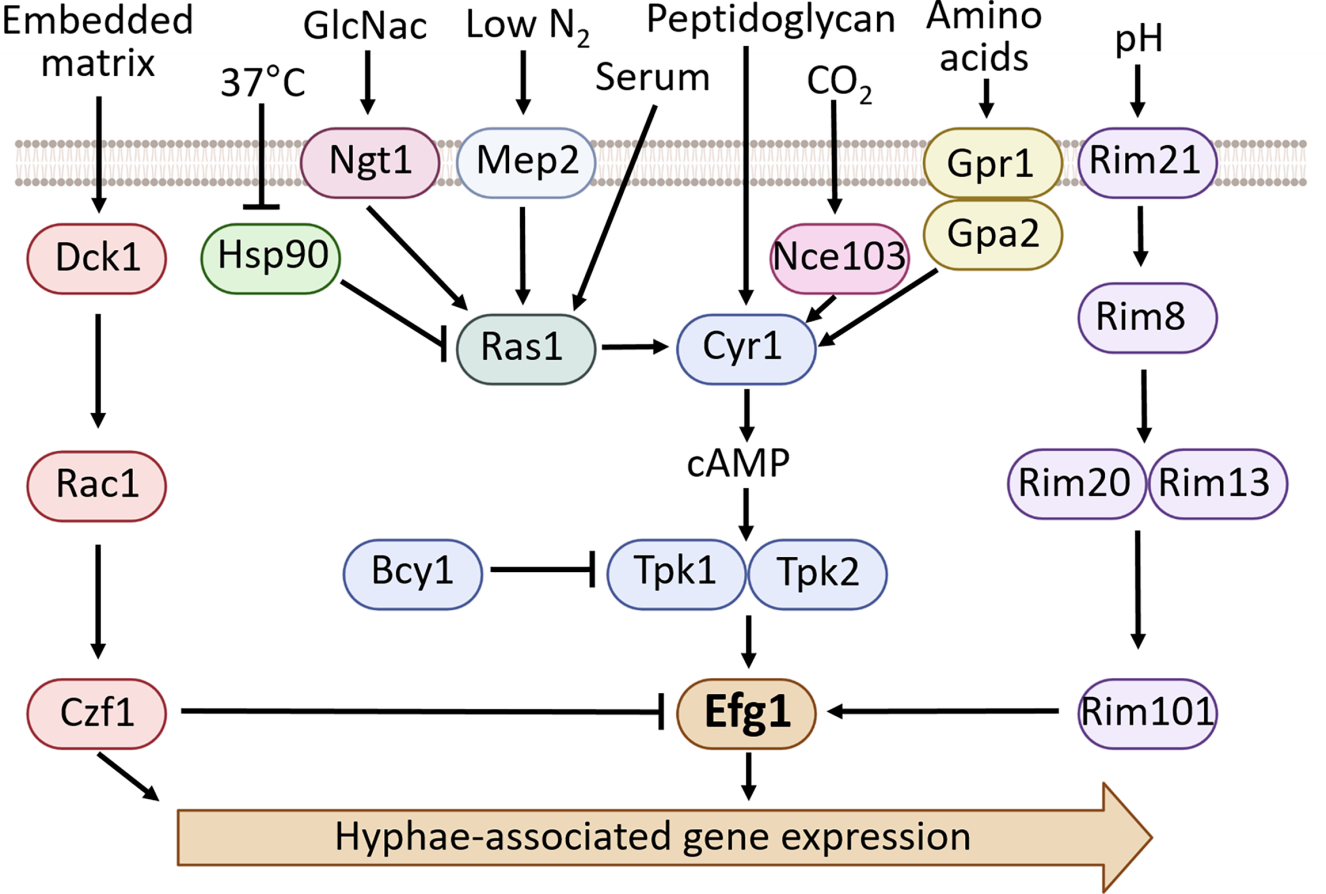
An overview of the environmental cues and factors that regulate hyphae formation in *C. albicans*, focusing on the signaling pathways involving Efg1 (Sudbery, 2011; Noble et al., 2017; Chen et al., 2020).

There are a wide variety of conditions that induce filamentation in *C. albicans* including factors that mimic the interstitial fluid environment such as serum (Taschdjian et al., 1960), 5% CO_2_ (Klengel et al., 2005), a neutral pH (Buffo et al., 1984), incubation at 37°C (Shapiro et al., 2009), and growth in tissue culture media (Sudbery, 2011). Factors that mimic the phagolysosome such as hydrogen peroxide and nitric oxide have also been shown to induce hyphal formation (Lo et al., 1997; Nasution et al., 2008; Uwamahoro et al., 2014; Koch et al., 2018). Other conditions and media that cause hyphal growth include the presence of N-acetylglucosamine (GlcNac) (Simonetti et al., 1974), growth within an embedded matrix (Brown Jr. et al., 1999), nutrient deprivation (Lu et al., 2011), nitrogen starvation (Lee et al., 2014), bacterial peptidoglycan (Xu et al., 2008) as well as Lee’s medium (Lee et al., 1975) and Spider medium among others (Liu et al., 1994). Although there are shared transcriptional patterns associated with hyphae formation under various conditions, different environmental cues trigger different signaling cascades to induce filamentation (Sudbery, 2011; Noble et al., 2017). Efg1 has been shown to both positively and negatively impact hyphae formation depending on the environmental cues and subsequent signaling cascades. **Figure 1** provides an overview of the environmental conditions and signaling cascades that result in Efg1-mediated induction of hyphae-associated gene expression.

The cAMP/PKA pathway is a significant signal transduction pathway for filamentation in *C. albicans* (**Figure 1**). In response to environmental cues such as low nitrogen, serum, CO_2_ or growth in Lee’s media/Spider media there is activation of Cyr1. Cyr1 is an adenylate cyclase which converts ATP to cAMP. The cAMP then binds to Bcy1, the regulatory subunit of the PKA complex (protein kinase A) (Giacometti et al., 2012; Ding et al., 2017). The interaction between cAMP and Bcy1 produces a conformational change that causes the dissociation of Bcy1 from the PKA catalytic subunits Tpk1 and Tpk2. Tpk1 and Tpk2 then phosphorylate target proteins, including Efg1. Epistasis analysis using overexpression of Efg1 in a *TPK2* deletion mutant restored filamentation on Spider media, while overexpression of Tpk2 in an *EFG1* deletion mutant did not restore filamentation under the same conditions (Sonneborn et al., 2000). Furthermore, mutation of Efg1 residue T208 (previously reported as T206) to a glutamic acid that mimics phosphorylation produces robust filamentation on Spider media, and also rescues filamentation in a *TPK2* deletion mutant (Bockmühl and Ernst, 2001). These results suggest that Efg1 functions downstream of Tpk2. Upon activation by the cAMP/PKA pathway, Efg1 binds to the promoter regions of hyphae associated genes, including several genes that encode for transcriptional regulators such as *EED1*, *TEC1* and *CRZ1*. Efg1 also binds to the *EFG1* transcript itself. (Lassak et al., 2011). In addition to binding transcripts directly, Efg1 has been shown to work in combination with Flo8 to regulate gene expression during the transition to hyphal growth in serum (Cao et al., 2006).

Efg1 has been observed within the nuclei of yeast cells under normal growth conditions and also upon hyphal induction. Following hyphal induction both the expression of *EFG1* and nuclear levels of Efg1 drop dramatically suggesting Efg1 may be important for the initial transition to hyphae but not continued hyphal growth in serum and Spider media (Tebarth et al., 2003; Bharucha et al., 2011; Saputo et al., 2014). Immediately following the addition of serum to induce filamentation, Efg1 is found bound to the *EFG1* promoter, and expression of *EFG1* declines dramatically. As such, Efg1 is likely acting as a negative autoregulator of its own expression under these conditions. (Lassak et al., 2011). This downregulation of *EFG1* is required for continued hyphal formation as overexpression of *EFG1* in the presence of serum prevented the formation of hyphae and instead produced yeast and pseudohyphae cells (Tebarth et al., 2003). It is important to note that *EFG1* expression levels demonstrate considerable cell-to-cell variability in standard YPD media (and in the host), therefore the changes in expression patterns that occur following a transition to hyphal induction conditions are representative of what is occurring at the population level (Pierce and Kumamoto, 2012).

In *C. albicans* pH is a strong regulator of morphology with a low pH environment restricting growth of *C. albicans* to yeast cell types while a neutral or alkaline pH is permissive to filamentation. Rim101 has been characterized as the primary regulator of pH dependent cellular responses by promoting expression of alkaline-associated genes while repressing acidity-associated genes under neutral pH conditions. Deletion of *RIM101* prevents hyphae formation under neutral pH conditions (Davis et al., 2000). Conversely, mutations that result in constitutive activation of Rim101 allow for filamentation at low pH. Deletion of *EFG1* in dominant active *RIM101* strains suppresses the overactive filamentation phenotype at low pH. This observation suggests that Efg1 functions downstream of Rim101 to promote filamentation in a pH dependent manner (Barkani et al., 2000).

In contrast to its widely described role as a positive regulator of filamentation, Efg1 also acts as a suppressor of filamentation under embedded and hypoxic conditions, as deletion of *EFG1* under these conditions causes an increase in filamentation (Giusani et al., 2002; Doedt et al., 2004; Mulhern et al., 2006). The increase in filamentation observed in *efg1*Δ/*efg1*Δ mutants under hypoxic conditions occurs at temperatures <37°C suggesting the repressive effects of Efg1 are temperature dependent (Setiadi et al., 2006). Czf1 is also involved in the filamentation pathway under hypoxic conditions with deletion of *CZF1* decreasing filamentation in an embedded matrix (Brown Jr. et al., 1999). Epistasis-based genetic models suggest that Czf1 may act to relieve Efg1 suppression of filamentation. Evidence to support this hypothesis can be found in the fact that Czf1 binds Efg1 in a yeast two-hybrid screen (Giusani et al., 2002). Additionally, changes in *CZF1* expression (overexpression or deletion) did not alter the hyperfilamentation phenotype of the *efg1*Δ/*efg1*Δ mutant in embedded conditions. This would be expected in a model where the effect of Czf1 on filamentation is dependent on suppression of Efg1 (Giusani et al., 2002). *EFG1* has also been implicated in the formation of chlamydospores under embedded conditions. These large, round cells can be found attached to suspensor cells and can be induced under specific conditions that include nutrient deprivation and low temperature combined with embedded conditions. Deletion of *EFG1* has also been shown to prevent chlamydospore formation despite the *efg1*Δ*/efg1*Δ mutant being hyperfilamentous under these conditions (Sonneborn et al., 1999b).

Under normoxic conditions expression of Efg1 is suppressed through interactions with Ifu5 (Rastogi et al., 2020) Following the transition to a hypoxic environment suppression of Efg1 by Ifu5 decreases, and as a result Efg1 expression increases. Under these hypoxic conditions Efg1 downregulates *CEK1* and *CPH1*, components of the *CEK1* MAP (Mitogen Activated Protein) kinase cascade required for filamentation (Desai et al., 2015). Together with Bcr1, Efg1 and Bcr1 work together to repress filamentation under hypoxic conditions with elevated CO_2_ levels (Desai et al., 2015). In addition to repressing filamentation under hypoxic conditions, Efg1 is also responsible for the induction of hypoxia-responsive genes as well as metabolic reprogramming under hypoxic conditions through changes in the expression of genes involved in unsaturated fatty acid metabolism (Setiadi et al., 2006). It is possible that the temperature and hypoxic dependent effects of Efg1 to repress filamentation may act to promote colonization of *C. albicans* within specific niche microenvironments of the host, however this hypothesis is in conflict with other evidence on the role of Efg1 in gut commensalism discussed below (Desai et al., 2015).

Many of the conditions used for examining filamentation *in vitro* mimic host conditions in an effort to identify and examine factors that influence *in vivo* filamentation. A comparison of transcriptomic profiles for multiple solid and liquid filamentation has identified some common filamentation response genes including *ALS3*, *HWP1, DCK1, IHD1*, and *RBT1*, however the transcriptomic profiles for filamentation differ dramatically under different filamentation inducing conditions (Azadmanesh et al., 2017). Additionally, as mentioned previously Efg1 has disparate roles in filamentation under different *in vitro* conditions. As a result *in vitro* filamentation may not be an accurate predictor of *in vivo* filamentation, depending on the type of filamentation inducing conditions used. Despite these caveats it does appear as though media that mimics the host environment, particularly RPMI solid media, is a more accurate predictor of virulence within the host than other forms of media tested (Azadmanesh et al., 2017). A study by Wakade et al. examined the differences between filamentation *in vitro* and *in vivo* for several genes associated with filamentation including *EFG1* across multiple clinical isolates. Comparison of the filamentation of the *efg1*Δ*/efg1*Δ mutant strain to the parental strain in RPMI + 10% serum demonstrated a decrease in filamentation in the *efg1*Δ*/efg1*Δ mutant across five of the six clinical isolates tested (the sixth strain, P75010, has significantly reduced filamentation compared to the other *C. albicans* clinical isolates and therefore the reduction in filamentation observed in the *efg1*Δ*/efg1*Δ mutant compared to the parental strain was not statistically significant). Similarly, intravital imagining of *efg1*Δ*/efg1*Δ mutants compared to the parental strains in a mouse ear model revealed a statistically significant decrease in filamentation in the *efg1*Δ*/efg1*Δ mutants among all clinical isolates tested (Wakade et al., 2021). These results suggest that *EFG1* does appear to be a central regulator of filamentation *in vivo* across multiple clinical isolates.

## Efg1 Is a Central Regulator of the Biofilm Transcription Factor Regulatory Network

In addition to being an important regulator of filamentation, Efg1 is one of the six core transcription factors that regulate biofilm formation in *C. albicans* (Nobile et al., 2012; Glazier et al., 2017). Biofilms are complex highly structured communities of fungi and/or bacteria. *C. albicans* biofilms are composed of yeast, pseudohyphae and hyphal cells surrounded in an extracellular matrix. Biofilm formation requires several steps that begin with the adhesion of yeast cells to a surface. Following adherence the yeast cells begin to proliferate producing both pseudohyphal and hyphal cells. The increase in cell density allows for the biofilm to develop 3D architecture, aided by the projections of hyphal cells and the synthesis of polysaccharide extracellular matrix. Once the mature biofilm has developed, yeast cells are dispersed to seed new sites of infection (Blankenship and Mitchell, 2006; Nobile and Johnson, 2015). The biofilm transcription factor regulatory network is composed of *BRG1, NDT80, ROB1, TEC1, BCR1*, and *EFG1* (Nobile et al., 2012; Nobile and Johnson, 2015). Transcription factors within this regulatory network control (either directly or indirectly) crucial aspects of biofilm architecture such as the production of adhesion proteins and extracellular matrix material, as well as the morphological transitions between cell types including hyphae formation, dispersal cells, and persister cells.

Given the critical role of Efg1 in filamentation, the biofilm formation defects in the *efg1Δ/efg1Δ* mutant are often attributed to the inability to form hyphae, as hyphae are required for the architectural stability of the biofilm. However, there is evidence to suggest that biofilm formation is not entirely dependent on filamentation. Deletion of both *CPH1* and *EFG1* results in a mutant that is unable to form hyphae yet still has some ability to bind to abiotic surfaces and form a biofilm entirely of yeast cells under specific environmental conditions (García-Sánchez et al., 2004). Similarly, overexpression of *ALS3* in the *efg1*Δ*/efg1*Δ mutant allows for the formation of yeast only biofilms on catheter material *in vitro*, suggesting that adhesion defects may prevent biofilm formation in the *efg1*Δ*/efg1*Δ deletion strain. Additional evidence that adhesion is required for biofilm formation can be found in the *BCR1* deletion mutant which has downregulation of several adhesin factors including *ALS1, ALS3* and *HWP1*, causing a biofilm formation defect despite being able to form hyphae (Nobile et al., 2006).

The adhesion of *C. albicans* to surfaces and the attachment of cells to one another requires several adhesins that are downstream targets of Efg1 including the adhesin proteins *ALS1, ECE1* and *HWP1* (Braun and Johnson, 2000; Fu et al., 2002; Nobile et al., 2012). Efg1 has also been shown to regulate both *ALS3* and *EAP1* under serum conditions (Leng et al., 2001; Li and Palecek, 2003; Argimón et al., 2007). Additionally, as mentioned previously, overexpression of *ALS3* in the *efg1*Δ*/efg1*Δ strain rescues biofilm formation on catheter material *in vitro* (Zhao et al., 2006). However, Efg1 was not observed to bind *ALS3* nor *EAP1* in a chromatin immunoprecipitation assay of biofilms formed with Spider media at 37°C (Nobile et al., 2012), despite both *ALS3* and *EAP1* being required for biofilm formation (Zhao et al., 2006; Li et al., 2007). It is possible that Efg1 binds to the promoter regions of *EAP1* and *ALS3* under specific environmental cues. Alternatively, the effects of Efg1 on *EAP1* and *ALS3* may be through indirect interactions with other target proteins. Addition evidence that contradicts a role for *EFG1* in adhesion can be found in flow cells assays measuring the adhesion of mutants grown in YPD to a silicone substrate (Finkel et al., 2012). In this assay, the *efg1*Δ*/efg1*Δ mutant has a mild, but not statistically significant decrease in adhesion as compared to wild type. These experiments were performed in YPD so it is possible that under different media conditions (including those that elicit biofilm formation) *EFG1* may have a greater impact on the adhesion process (Finkel et al., 2012).

**Table S1** includes a list of transcripts with twofold change in expression in the *efg1*Δ*/efg1*Δ mutant compared to wild type under biofilm conditions that also have been identified as having promoter regions that Efg1 binds to (Nobile et al., 2012). These represent transcripts in which Efg1 is likely directly regulating the expression of. Key components of biofilm formation including *ECE1, BRG1, ALS1, NDT80, BCR1* and *TEC1* are all downregulated in the absence of *EFG1*, likely contributing the decrease in biofilm formation observed in the *efg1*Δ*/efg1*Δ mutant (Nobile et al., 2012). At least a portion of the phenotype that is produced by a decrease/loss of *EFG1* is likely dependent on the activity of Tec1 downstream of Efg1, as overexpression of *TEC1* is able to rescue biofilm formation in an *efg1*Δ*/EFG1* heterozygous deletion strain (Glazier et al., 2017).

The biofilm transcription factor regulatory network has been extensively studied in the SC5314 background (Fox and Nobile, 2012; Nobile et al., 2012; Fox et al., 2015; Nobile and Johnson, 2015; Glazier et al., 2017; Mancera et al., 2021). These studies have characterized a fragile network *in vitro* whereby deletion of a single transcription factor within the network disrupts biofilm formation (Glazier and Krysan, 2018). However recent work comparing the biofilm transcription factor regulatory network in SC5314 to other clinical isolates of *Candida albicans* has revealed dramatic differences in regulatory circuits that govern biofilm formation between strains (Huang et al., 2019). All of the clinical isolates studied had decreased biofilm formation upon deletion of *EFG1*, providing support for *EFG1* as an essential regulator of biofilm formation in all *C. albicans* strains. Consistent with what is observed in SC5314, deletion of *EFG1* in the 4 clinical isolates tested also resulted in decreased expression of *ALS1* and *BRG1*. However deletion of *EFG1* decreased expression of *ECE1* in only three of the four clinical isolate strains studied. Similarly, only two of the four clinical isolate strains had a decrease in *TEC1* expression when *EFG1* was deleted (Huang et al., 2019). Therefore although Efg1 is a central regulator of biofilm formation across all isolates tested, is appears to regulate different subsets of the transcripts required for biofilm formation among the various clinical isolates.

In this context, the fragility of the biofilm network observed in SC5314 raises an interesting evolutionary question around how such a fragile transcription factor regulatory network could have evolved. The SC5314 biofilm transcription factor regulatory network has a lower than expected number of genes with close homologs in other *Candida* species (Mancera et al., 2021). This observation combined with the lower than expected frequency of Ndt80 and Efg1 consensus elements in other closely related species suggests the *C. albicans* biofilm regulatory network may be a more recent evolutionary adaptation. Additionally, ChIP-chip analysis of the transcription factor binding sites to target genes promoters has identified larger than expected promoter regions, which may facilitate relatively quick incorporation of new target genes into the regulatory network (Nobile et al., 2012; Mancera et al., 2021). Based on these observations it is possible that some of the disparities between the different clinical isolates are due to the incorporation of targets into the biofilm transcription factor regulatory network that do not directly contribute to biofilm adaptation. These target genes are then maintained within in a given clinical isolate due to neutral (non-adaptive) selection. However this hypothesis does not explain why the core transcription factor regulatory networks between the clinical isolates also seems fundamentally altered. The variability in biofilm regulatory circuitry in clinical isolates reinforces the need to study additional *C. albicans* strains to determine the commonalities between the regulatory circuitry patterns that govern biofilm formation across multiple isolates. This variability also highlights the dynamic capacity for *C. albicans* to maintain robust biofilm formation using different transcription factor network architecture in different strains (Huang et al., 2019).

## Efg1 Is Required for Transition to, and Maintenance of, the White State

In addition to yeast cells, pseudohyphae and hyphal morphology, *C. albicans* is also able to transition while in the yeast confirmation between white, gray, and opaque phenotypes. Characterized by their colony appearance, the white, gray, and opaque states have specific cellular morphology, metabolic programing, cell wall traits, as well as effects on both virulence and commensalism. The transcription factor regulatory network that controls white/gray/opaque phenotype switching includes Efg1, Wor1, Wor2, Wor3, Ahr1 and Czf1 (Hernday et al., 2013). Efg1 is highly expressed in the white cell state. Overexpression of *EFG1* causes opaque state cells to transition to the white state (Sonneborn et al., 1999a). Conversely, in opaque cells, *EFG1* is expressed at low levels. Deletion of *EFG1* produces cells that form an intermediate gray state in **a**/**a,** α/α cell types as well as some **a**/α clinical isolates (Sonneborn et al., 1999a; Srikantha et al., 2000; Zordan et al., 2007; Xie et al., 2013; Liang et al., 2019; Park et al., 2019; Park et al., 2020). Once in the gray state, *efg1*Δ/*efg1*Δ mutants in **a**/**a** and α/α MLT strains have a high rate of transition to the opaque state. Hemizygosity of *EFG1*, which is often seen in clinical isolates, produces cell types that can switch to the intermediate gray state and then undergo additional conversion to the opaque state depending on environmental conditions and selection pressures (Liang et al., 2019).

In white cells, the increased expression of Efg1 allows for Efg1 to repress expression of *WOR1*, the master regulator of the opaque state, thereby maintaining the cells in the white state. The repression of *WOR1* by Efg1 is direct, as Efg1 binds to the *WOR1* promoter in white cells (Hernday et al., 2013). Brg1 has also been identified as binding to the *WOR1* promoter under biofilm conditions (Nobile et al., 2012). Evidence that deletion of either Brg1 or Efg1 in a heterozygous *MTL* **a**/α strain allows for white to opaque switching suggests that Brg1 may also repress *WOR1* expression in conjunction with Efg1, however further research is necessary (Xie et al., 2013). Efg1 also acts with Ahr1 (Zcf37) to repress *WOR2* (Zordan et al., 2007; Sriram et al., 2009; Wang et al., 2011). *WOR2* is required for maintenance of the opaque state, with *WOR1* and *WOR2* forming a positive feedback loop promoting the opaque cell type (Zordan et al., 2007; Sriram et al., 2009). Ahr1 binds to the promoter of *EFG1* and *WOR2*, therefore in addition to Efg1 acting in concert with Ahr1 to downregulate *WOR2*, Ahr1 may also regulate *EFG1* expression (Hernday et al., 2013). In summary Efg1 represses *WOR1* both directly by binding to the promoter of *WOR1* and indirectly through repression of *WOR2.*

In the opaque state *EFG1* expression is downregulated by both Czf1 and Wor1. Czf1 represses *EFG1* expression in opaque cells, which in turn results in increased expression of *WOR2*, facilitating the transition to the opaque state (Sriram et al., 2009; Hernday et al., 2013). Wor1 also binds to the promoter of *EFG1* acting as a repressor of *EFG1* expression (Zordan et al., 2007; Pujol et al., 2016). A transcriptomic analysis comparing white and opaque cell types identified a shortening of the 5’UTR (untranslated region) of *EFG1* during the opaque state with the transcription start site near the Wor1 binding site. This data suggests that Wor1 may direct changes in the transcription start site producing a shorter 5’ UTR of *EFG1* while cells are in the opaque state (Tuch et al., 2010). 5’UTR modifications are known to influence ribosomal recruitment and therefore translation. It is possible that the shorter 5’ UTR of *EFG1* during the opaque state may modify post-translational regulation of the *EFG1* transcript. In particular, the shorter 5’UTR may result in changes in ribosomal recruitment that decrease translation of Efg1. **Table S1** includes microarray results comparing *efg1*Δ/*efg1*Δ mutant and wild type transcriptomic profiles in both white and opaque states (Hernday et al., 2013). The data has been filtered to only include genes with Efg1 bound to the promoter region that also demonstrate a twofold or greater change in gene expression when *EFG1* is deleted.

## Efg1 Has Opposing Functions in Commensalism and Invasive Infection

A similar dynamic between Efg1 and Wor1 observed in white/gray/opaque switching is also found in the regulation of commensalism within the gut. In the mouse gastrointestinal (GI) tract Wor1 acts as a driver for the GUT phenotype, facilitating commensalism within the gastrointestinal tract, with deletion of *WOR1* causing decreased fitness in a commensalism model (Pande et al., 2013). Conversely, Efg1 inhibits commensalism with the *efg1*Δ/*efg1*Δ mutant displaying a gray phenotype (similar to the GUT phenotype) and increased commensalism, outcompeting wild type cells in a mouse model of gastrointestinal candidiasis (Pierce and Kumamoto, 2012; Pande et al., 2013; Cottier and Hall, 2020). Calling card-seq, a method by which transcription factor binding sites can be mapped using transposons *in vivo* during GI colonization, show that Czf1 binds *EFG1* (Witchley et al., 2021). Additionally gut colonization of the double deletion mutant *efg1*Δ/*efg1*Δ and *czf1*Δ*/czf1*Δ produces the same phenotype as *efg1*Δ/*efg1*Δ alone. These results suggest the effect of Czf1 on commensalism is *EFG1* dependent, supporting a similar transcription factor regulatory network to what is seen in white/gray/opaque switching where Czf1 regulates *EFG1* expression (Witchley et al., 2021).

It is speculated that Efg1 likely inhibits commensalism within the gastrointestinal tract through multiple mechanisms. The first method is by inhibiting Wor1, which as mentioned previously, is the central regulator of the GUT phenotype and required for commensalism. Efg1 also represses expression of other transcripts required for gut colonization including *CHT2* which encodes a GPI-linked chitinase (Witchley et al., 2021). The third mechanism is through upregulating hyphal specific genes, particularly *SAP6* which encodes a pro-inflammatory aspartyl protease. Sap6 has been hypothesized to trigger a localized immune response that restricts invasive hyphal growth in the GI tract (Pietrella et al., 2010; Witchley et al., 2019; Witchley et al., 2021). Similar dynamics are seen when other transcription factors that promote hyphal formation are deleted, including *BRG1, ROB1, TEC1*, and *UME6*. Deletion of any one of these transcription factors produced mutants with increased commensalism as compared to wild type. It is worth noting that hyphae can still be found in parts of the gut, particularly the large intestine and cecum, where several drivers of filamentation, N-acetylglucosamine, hypoxia, and high levels of CO_2_ likely promote hyphae formation (Witchley et al., 2019).

Evidence for a multifaceted role of Efg1 in commensalism is supported by the GI fitness of the *efg1*Δ/*efg1*Δ *wor1*Δ*/wor1*Δ double mutant which displays an intermediate GI fitness between *efg1*Δ/*efg1*Δ and *wor1*Δ*/wor1*Δ single deletion mutants. In this double deletion mutant the loss of Wor1, the driver of the GUT phenotype, decreases commensalism. Yet the mutant presumably is still has higher levels of *CHT2* and lower levels of the hypha specific transcripts such as *SAP6*, similar to the *efg1*Δ/*efg1*Δ mutant. The combination of factors likely results in the intermediate GI fitness (Witchley et al., 2021). A list of genes differentially regulated between wild type and the *efg1*Δ/*efg1*Δ during gut commensalism can be found in **Table S1** (Witchley et al., 2021). Transcripts were filtered by a twofold change in expression compared to wild type, and the ability of Efg1 to bind the promoter region of the gene.

There is conflicting evidence for the role of Efg1 in oropharyngeal candidiasis. In a gnotobiotic pig model of oropharyngeal candidiasis *efg1*Δ/*efg1*Δ mutants are able to form hyphae (Riggle et al., 1999). Similarly, deletion of *EFG1* produces a variable fungal burden in an oropharyngeal candidiasis mouse model with the *efg1*Δ/*efg1*Δ mutants still able to form hyphae *in vivo* (Solis et al., 2022). However in another oropharyngeal candidiasis mouse model deletion of *EFG1* decreased fungal burden with the *efg1*Δ/*efg1*Δ mutant strain forming yeast and short pseudohyphae (Park et al., 2005). It is possible that the differences in morphology and virulence may be attributable to differences in the host model (male CD-1 compared to female BALB/c mice) and/or strain (SN background compared to CAI). Future studies are required to determine whether the variability in the role of *EFG1* in OPC virulence is due to diversification of the transcription factor regulatory network that governs OPC, as seen in the biofilm regulatory network which includes *EFG1* (Huang et al., 2019), or whether the differences observed are due to nuances in the experimental techniques and/or models.

Much of the work done on interactions between the host immune system and *C. albicans* has focused on the *cph1*Δ/*chp1*Δ *efg1*Δ/*efg1*Δ yeast locked strain, and it is therefore hard to deduce the role of Efg1 versus Cph1. However *EFG1* is required for the transition to hyphae in both macrophages and neutrophils (Korting et al., 2003; McKenzie et al., 2010; Wellington et al., 2012). Deletion of *EFG1* results in cells that are unable to trigger macrophage lysis as measured by LDH release (Wellington et al., 2012). Studies on macrophage activation following engulfment of *efg1*Δ/*efg1*Δ mutants have shown that these mutants are unable to activate caspase 1, trigger the ROS response, and have macrophage responses with lower levels of IL-1β, TNF-α, IL-4, and IL-23 cytokine production. Changes in immunogenicity of the *efg1*Δ/*efg1*Δ mutants compared to wild type is likely due to a combination of changes on the surface of the cell including changes in cell wall architecture (Wellington et al., 2012; Zavrel et al., 2012).

Lower levels of *EFG1* results in a fitness advantage within the gut when compared to cells with higher levels of *EFG1* expression (Pierce and Kumamoto, 2012; Pande et al., 2013). It is perhaps not surprising then that examination of clinical isolates has identified several strains that were naturally hemizygous for *EFG1* (Liang et al., 2019). Passaging through the mouse GI tract results in decreased expression of *EFG1* with clinical isolates that are hemizygous for *EFG1* further transitioning into an *EFG1* null state, supporting the GI fitness advantage conferred by loss of *EFG1* expression (Liang et al., 2019). Similarly, evolution studies using SC5314 passage through the GI tract of mice treated with antibiotics produced mutants homozygous for *de novo* non-synonymous mutations in *EFG1*, again suggesting a selective pressure whereby loss of *EFG1* increases fitness in the gastrointestinal tract (Tso et al., 2018). Although decreasing *EFG1* expression produces a fitness advantage within the gastrointestinal tract, *EFG1* is required for invasive disease with *efg1*Δ/*efg1*Δ mutants being avirulent in a tail vein injection mouse model (Lo et al., 1997; Pierce and Kumamoto, 2012; Pande et al., 2013). *EFG1* expression levels are also dependent on the immune status of the host, with *EFG1* expression decreasing in the gastrointestinal tract in an immunocompromised mouse model (Pierce and Kumamoto, 2012). Taken together, these observations suggest *EFG1* expression is dynamic, highly variable, and that control of Efg1 levels is essential to the survival of *C. albicans* as both a pathogen and commensal. It is likely that within the host different populations of *C. albicans* express varying levels of *EFG1* in response to environmental factors present within the niche site, and that the selection pressures within these sites select for mutations that results in changes to *EFG1* expression which confer increased fitness under those conditions. This natural genetic diversity of *C. albicans* within a single host has been proposed to allow rapid adaptation in response to changes in the host environment (Sitterlé et al., 2019).

## Efg1 Has Both Shared and Distinct Targets Under Different Environmental Conditions

**Figure 2:**
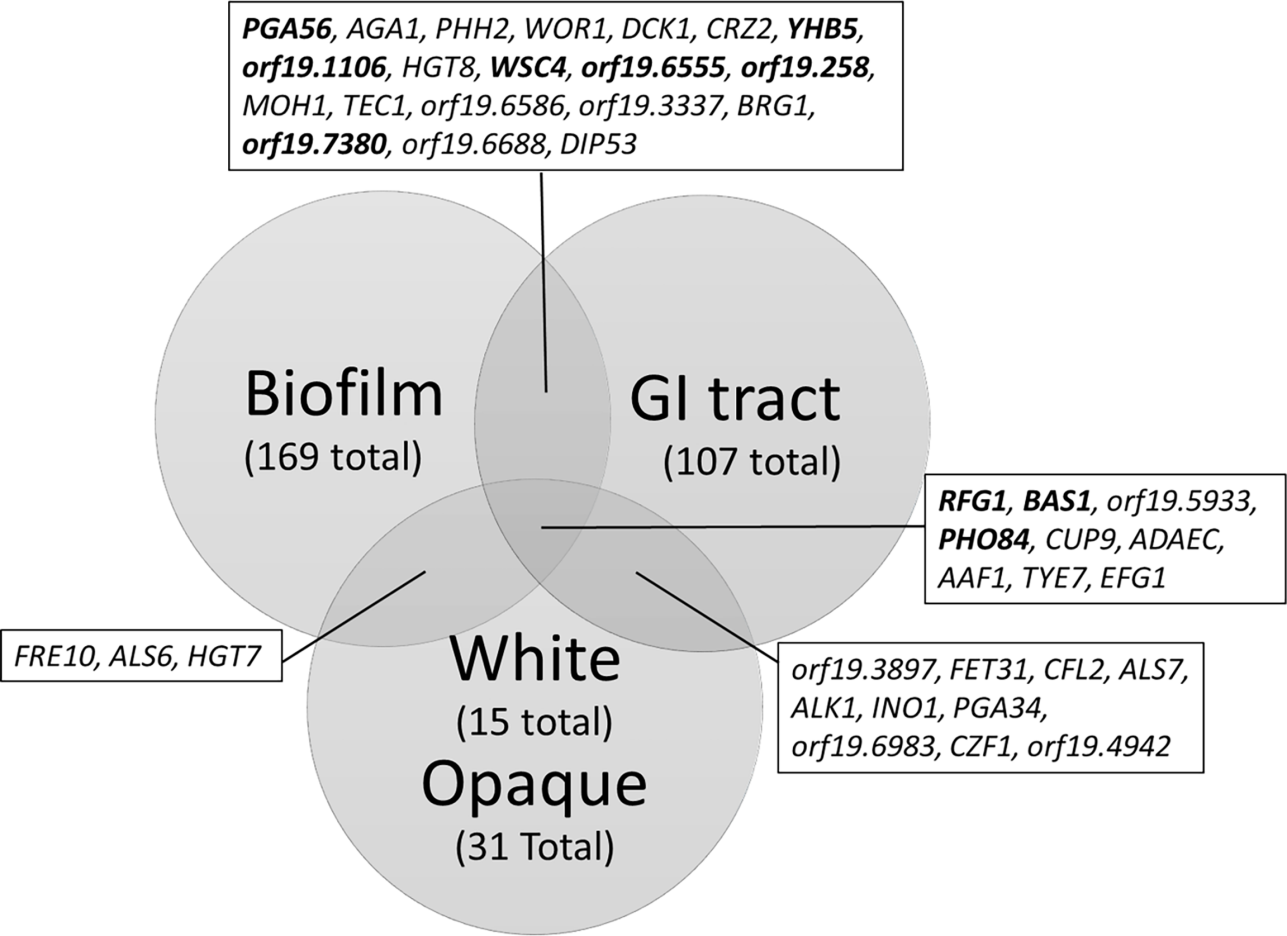
Venn Diagram of Efg1 targets during biofilm formation, gut commensalism, and either the white or opaque state. Transcriptomic studies comparing the *efg1*Δ/Δ deletion mutant and wild type strains under the conditions listed were used to determine transcripts with twofold changes in expression upon deletion of *EFG1.* Transcripts were then filtered by whether Efg1 is able to bind to the promoter regions of the encoding gene to create a list of Efg1 targets with differential expression upon deletion of *EFG1.* Bold identifies transcripts that are differentially regulated (upregulated in one condition, downregulate in another). Data adapted from (Nobile et al., 2012; Hernday et al., 2013; Witchley et al., 2021).

Efg1 has been observed to have different consensus elements and different targets depending on environmental conditions, likely as the result of changes in phosphorylation patterns and/or interactions with other transcription factors. These differences in targets allow for Efg1 to function as a regulator of the very different morphological programs required for of gut commensalism, filamentation, biofilm formation, and the white state. By compiling the information on Efg1 binding to targets along with transcriptomic changes in *EFG1* mutants we can compare the functionally relevant Efg1 targets differentially regulated in gut commensalism, biofilm formation, and transition between white and opaque states (**Figure 2**) (Nobile et al., 2012; Hernday et al., 2013; Witchley et al., 2021). Targets were included if they exhibit both Efg1 binding to the promoter regions and a twofold change in gene expression when *EFG1* was deleted. The core set of target genes that are shared under all three conditions include *RFG1, BAS1, PHO84, CUP9, ADAEC, AAF1, TYE7*, and *EFG1*. Of the share target genes, *RFG1, BAS1, CUP9*, and *TYE7* all encode transcription factors, consistent with the previously describe roles of Efg1 in multiple transcription factor regulatory networks (Nobile et al., 2012; Hernday et al., 2013; Rodriguez et al., 2020). Efg1, as previously mentioned, has been showed to bind its own promoter region and appears to be autoregulatory in all three conditions. The overlap in targets is highest between biofilm formation and commensalism, which may reflect similar roles of Efg1 in hyphal morphogenesis. While the overlap in targets between commensalism and the white/opaque state may reflect similarities in the *efg1*Δ/*efg1*Δ mutants to both GUT and gray/opaque phenotypes.

## Conclusions

In the roughly 25 years since *EFG1* was first characterized in *Candida albicans*, *EFG1* has been explored in nearly all contents of *C. albicans* biology. Despite its popularity, or perhaps because of its popularity, several key questions remain with regard to Efg1 regulation and activity. One key question is relates to the activation and phosphorylation of Efg1 and whether differences in the consensus elements are governed by changes in phosphorylation status of Efg1, context specific interactions with other protein partners, or both. Another relatively unexplored aspect of *EFG1* is the post-transcriptional regulatory processes that control *EFG1* expression. Finally, given that *EFG1* expression within the host and Efg1 function in the regulatory network for biofilm formation is variable between clinical isolates, can we better understand the content of the changes in *EFG1* expression/function between strains to gain a more comprehensive understanding of *C. albicans* pathogenesis as a whole?

## Author Contributions

The author confirms being the sole contributor of this work and has approved it for publication.

## Funding

ACIS funding through Niagara University.

## Conflict of Interest

The author declares that the research was conducted in the absence of any commercial or financial relationships that could be construed as a potential conflict of interest.

## Publisher’s Note

All claims expressed in this article are solely those of the authors and do not necessarily represent those of their affiliated organizations, or those of the publisher, the editors and the reviewers. Any product that may be evaluated in this article, or claim that may be made by its manufacturer, is not guaranteed or endorsed by the publisher.

## Supplementary Material

Supplementary Table 1 Efg1 targets with two fold change upon deletion of *EFG1.* **(A)** Biofilm- Changes in RNA expression between the *efg1*Δ/Δ deletion mutant and wild type under biofilm formation conditions (Spider medium at 37°C in 6-well polystyrene plates) determined by RNA sequencing. Efg1 binding sites determined by chromatin immunoprecipitation tiling microarray (ChIP-chip) using Efg1-myc (Nobile et al., 2012). **(B)** White/opaque state- changes in RNA expression between the *efg1*Δ/Δ deletion mutant and wild type using gene expression microarray of cells in mid-log phase in synthetic complete media, Efg1 binding sites determined by ChIP-chip using Efg1 specific polyclonal antibodies (Hernday et al., 2013). **(C)** Gastrointestinal tract- changes in RNA expression between the *efg1*Δ/Δ deletion mutant and wild type in a commensalism mouse model within the gastrointestinal tract measured by RNA seq. Efg1 binding sites determined using Calling Card-seq with Efg1 fused to PiggyBac transposase (Witchley et al., 2021).
